# Two-step verification of brain tumor segmentation using watershed-matching algorithm

**DOI:** 10.1186/s40708-018-0086-x

**Published:** 2018-08-14

**Authors:** S. M. Kamrul Hasan, Mohiuddin Ahmad

**Affiliations:** 1 Department of Electrical and Electronic Engineering, Khulna University of Engineering Technology (KUET), Khulna, 9203 Bangladesh; 2 Present Address: Chester F. Carlson Center for Imaging Science, Rochester Institute of Technology (RIT), Rochester, NY 14623 USA

**Keywords:** Brain tumor segmentation, Median filter, Magnetic resonance imaging, SIFT algorithm, Status checking, Topology, Watershed-matching algorithm

## Abstract

Though the modern medical imaging research is advancing at a booming rate, it is still a very challenging task to detect brain tumor perfectly. Medical imaging unlike other imaging system has highest penalty for a minimal error. So, the detection of tumor should be accurate to minimize the error. Past researchers used biopsy to detect the tumor tissue from the other soft tissues in the brain which is time-consuming and may have errors. We outlined a two-stage verification-based tumor segmentation that makes the detection more accurate. We segmented the tumor area from the MR image and then used another algorithm to match the segmented portion with the ground truth image. We named this new algorithm as watershed-matching algorithm. The most promising part of our model is the status checking of the tumor by finding the area of the tumor. Our proposed model works better than other state-of-the art works on BRATS 2017 dataset.

## Introduction

**Fig. 1:**
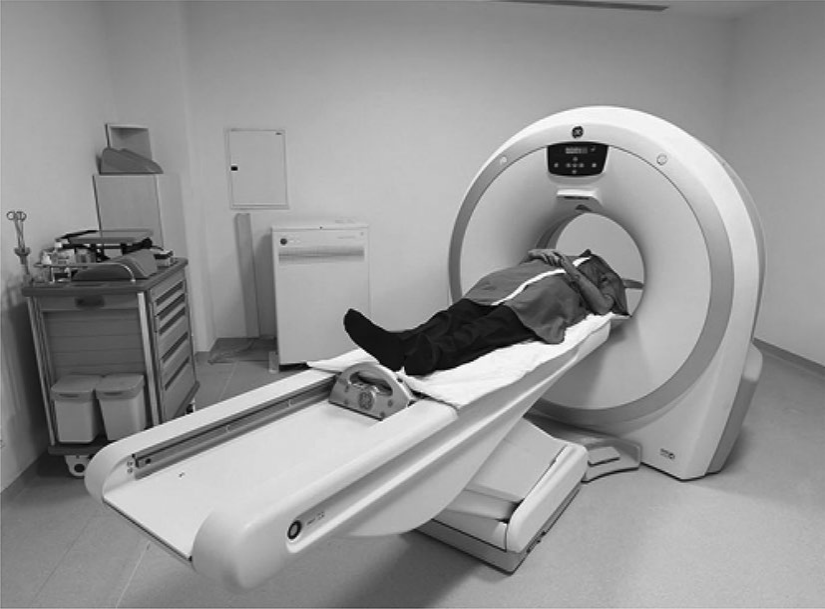
Example of magnetic resonance imaging (MRI) scanner setup in an authorized private medical hospital producing high-quality images of human brain. The motorized bed is moved inside the oval shaped scanner, and the head is scanned

Brain tumor is a mass or growth of abysmal tissues which originate in the brain itself or in the tissues such as meninges, pituitary glands, pineal gland, skull and neurons. Many different types of brain tumor exist. Some are cancerous which are called malignant, and some are noncancerous or benign. The most common and deadliest brain tumor type is gliomas with a subset known as glioblastoma ranging from slow growing low-graded tumors to high-graded malignant tumors. According to American Cancer Organizations, about 80,000 people are newly diagnosed with cancer per year around USA with 16,000 people dying from cancer. Of the cancer patients, approximately 32% are diagnosed with the malignant type brain tumor with a 5-year survival rate of 5.3%. As these tumors normally stay in the posterior cranial fossa of human brain so, it is difficult to detect it manually. Human brain has five types of soft tissues: white matter (WM), gray matter (GM), cerebrospinal fluid (CSF), edema and tumor tissue. Different tissues look different, and these differences can effectively be observed in the MRI scanning sequences which magnetize and demagnetize the hydrogen component of our human brain. Greater the hydrogen component, brighter the image. That is why for the high-graded gliomas (HGG) cases, necrosis and tumor tissues are delineated easily due to those hydrogen components. However, for the low-graded (LGG) cases, it is even difficult to delineate the tumor tissue. We used MRI for the assessment purpose. Brain soft tissues are easily differentiated in MR images unlike CT scan or ultrasonic or other images. A pictorial view of MR images taken from the MRI scanner is shown in Fig. 1.

## Literature review

Segmentation is the fundamental step in medical image analysis. Though past researchers have prepared their research, still now it is a vast research field because of the variation of the data of MRI. The authors in [1] use watershed segmentation and EM–GM algorithm for segmenting brain tumor. But they did not mention any potential dataset. Similar type research was approached by the authors in [2] who use support vector machine classifier to classify the tumor from the normal tissue. But, due to the fragile training set and not a better technique of feature extraction, their algorithm cannot robustly classify tumor. Some authors had tried scale-invariant feature transform (SIFT) algorithm to find the feature points and match the tumor region. As this algorithm finds the feature points in the high-cluster region, sometimes they misclassify the normal tissue as high-cluster tumor tissue [3]. Besbes et al. [4] introduced a model together with discrete Markov random field (MRF) for the segmentation of brain tumor. But the parameter estimation and computing probability for this method are very difficult. Shen et al. [5] introduced traditional fuzzy C-means (FCM) clustering algorithm. But it is prone to noise that may affect the pixel intensities and may have improper segmentation. Chen et al. [6] applied K-mean clustering and knowledge-based algorithm for biomedical image segmentation. But it only takes into consideration the image intensity, thereby not producing adequate outputs in noisy images. Many efforts have explored artificial neural network (ANN) [7]. Edge-based segmentation techniques cannot work well due to having inherent speckle noise and texture characteristics.

In addition, K-nearest neighbor (KNN) [8], support vector machine (SVM) [9], Bayesian algorithm, hidden Markov model, conditional random field [10], high-dimensional features with level set [11] are different segmentation algorithms. Unsupervised algorithms start to evolve in recent days [12, 13], albeit they are still in the early stages with non-autonomous.

We propose a model that has two levels of authentication system to detect tumor. We named it as the WM (watershed-matching) algorithm. For segmenting the tumor region, we used the classical watershed algorithm and then use SIFT (scale-invariant feature transform) algorithm for matching the segmented region with the original image. For calculating the volume of the tumor, we used a very different technique this time. We had developed an algorithm that will use the help of Freesurfer software to find the cortical thickness, and then, we will use this model to build a volume of the tumor and then can differentiate between the benign and malignant type.

Rest of the paper is organized as follows: The methodology of our work is reported in Sect. 3. In Sect. 4, we outline our experimental results. Finally, in Sect. 5, we draw a conclusion.

## Proposed methodology

**Fig. 2:**
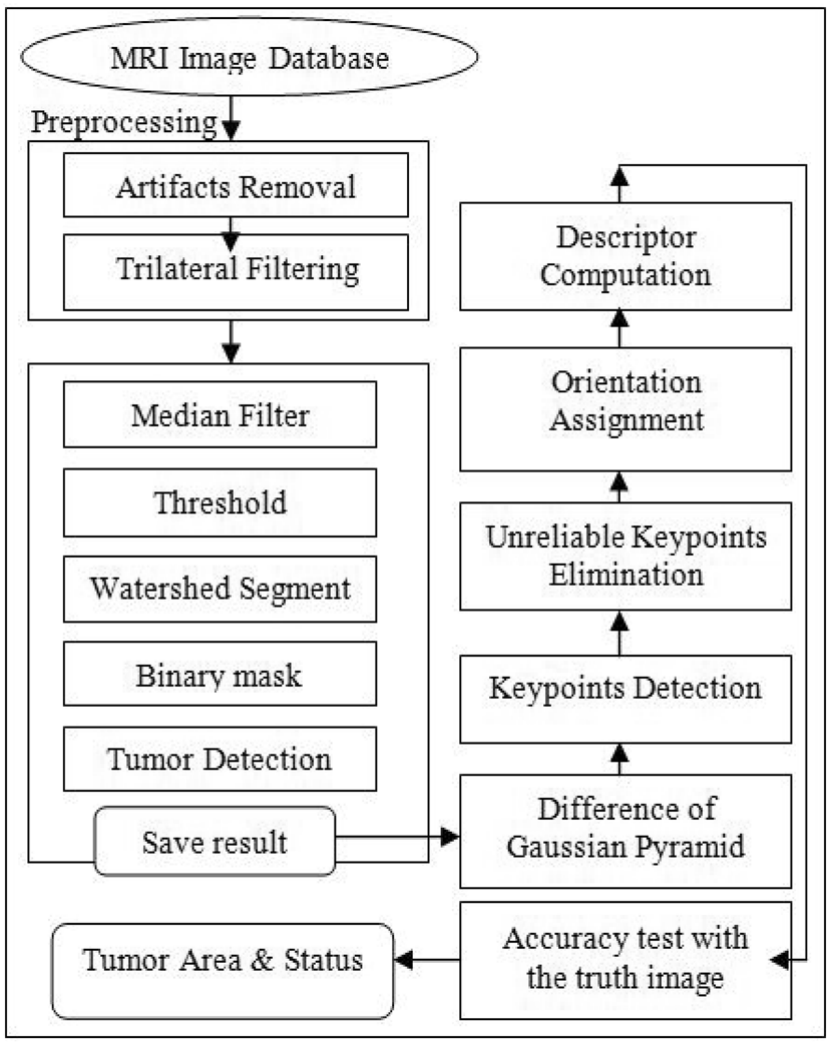
Block diagram of the watershed-matching algorithm. The complete paper is expounded on the above flowchart

The purpose of our segmentation technique is to segment brain tumor area from the MR images and giving the status of the brain tumor for diagnosis. Our proposed methodology is shown in Fig. 2.

### Image database

We used BRATS 2012 dataset that has multicontrast MR scans of 30 glioma patients, out of which 20 have been acquired from high-grade (anaplastic astrocytomas and glioblastoma multiform tumors) and 10 from low-grade (histological diagnosis: astrocytomas or oligoastrocytomas) glioma patients that had been manually annotated with two ground truth tumor labels (edema and core) by a trained human expert. The training data also contained simulated images for 25 high-grade and 25 low-grade glioma subjects with the same two ground truth labels. For each patient, multimodal (T1, T2, FLAIR and post-gadolinium T1c) MR images are available. I will use the BRATS 2013 test set that has multicontrast 10 high grades. The Leaderboard set has 11 + 10 high-grade glioma patients. It has also multimodal T1-weighted, T2-weighted, T2/FLAIR and post-gadolinium T1-weighted MR images. We have collected the tumor database from the MICCAI 2012 Challenge on Multimodal Brain Tumor Segmentation.

### Noise removal of input image

In this stage, we used the modified version of the bilateral filter for noise removal. Bilateral filter works very good for smoothening the steplike edge features. But the main problem with this filtering is that it only works well when the gradient changes are not very high. High-gradient changes have some outliers, and the window cannot detect those outliers. The modified version of this bilateral filtering is the trilateral filter that has tilted window to track the high-gradient regions. It works as the same way as the bilateral filtering works. It finds the gradient changes, but the difference is that it finds the skewed gradient. It is an extension to the bilateral filter [14]. Images corrupted with impulse noise can be removed by trilateral filter [15]. We used this filter for de-noising mixed noise and for image restoration. This filter smoothens the edges of the image and remains the details of the images fixed. It considers the nearby pixel information with the help of very narrow spatial window and needs a few iteration processes than bilateral filtering. It reduces the standard deviation and variance from the original image.

### Segmentation method

**Fig. 3:**
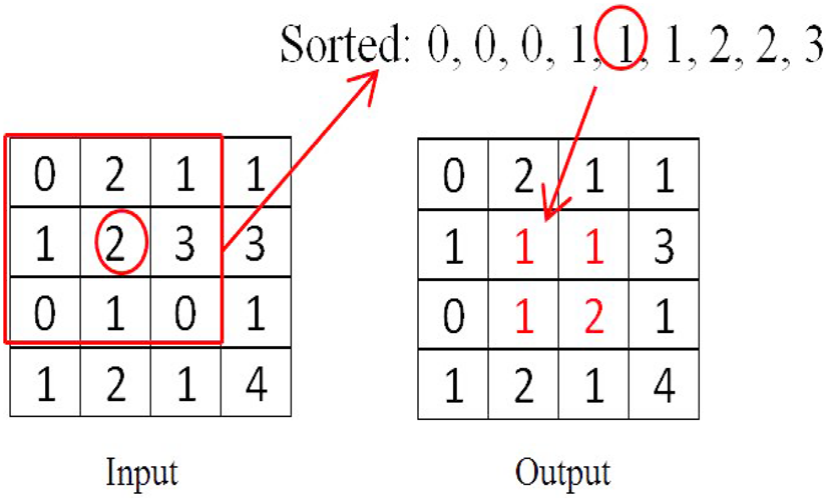
Example of a median filter using a 3 × 3 window. The pixel values inside the window are sorted, and then, find the median value and put the value in the middle of that window, thus keeping this process from left to right and then top to bottom

We used the watershed segmentation algorithm (WSA) for segmenting the tumor region because it provides a very good segmentation result, and at the same time, it is computationally less complex. Before applying watershed algorithm, we apply median filter to remove the high-frequency components from the MRI without disturbing the edges while reducing random and impulse noise. This filter replaces the pixel value with the median of those values, and to do this, all the pixel values are sorting in the ascending order from the neighborhood, and then, the pixel is replaced with the median value. Figure 3 illustrates an example. The median filter is robust than the average filter. It does not create any unrealistic pixel values, and hence, it is much better at preserving sharp edges than the average filter [16, 17]. We used 3 × 3 median filter kernel for removing any salt and pepper noise or pickle removal. We implemented the median filtering from the scratch. To preserve the similar pixel value as the input image, we used the zero padding so that the edge pixels are being filtered properly and the output would be the similar size as the input image. This zero padding can preserve the pixel value as the input.

#### Watershed segmentation algorithm (WSA)

To understand the watershed algorithm, we can think of a grayscale image as geological landscape as a metaphor where the watershed means the dam that divides the area by river system.

**Fig. 4:**
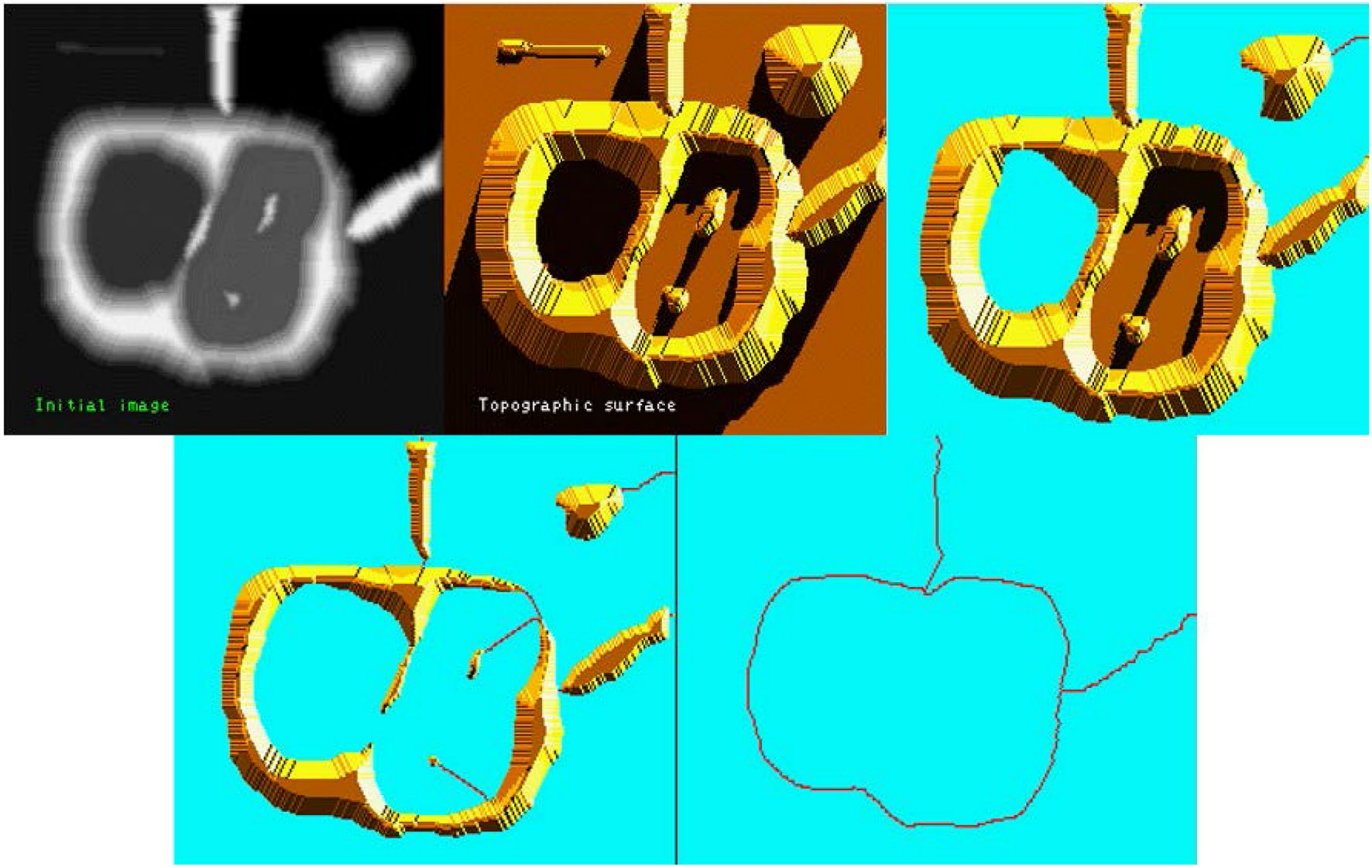
Core watershed segmentation algorithm (from left to right; top to bottom). Input image, topological surface, water flooded in the surface, water continues to flood, segmentation after completion of flooding

In watershed transform, an image can be regarded as a topological surface, where the value of *I*(*x*, *y*) corresponds to heights. From Fig. 4, water would collect in one of the two catchment basins. Water falling on the watershed ridge lines separating the two basins would be equally likely to collect into either of the two catchment basins. WSA then find the basins and the ridge lines in an image.

Let consider the algorithm: A hole is punched at each regional local minimum, and the entire topology is flooded from below by letting the water rise through the holes at a uniform rate. Pixels below the water level at a given time are marked as flooded. When the water level rises, the flooded region will also grow. When this occurs, the algorithm will construct a one-pixel-thick dam that separated the two regions. The flooding continues until the entire image is segmented into separate catchment basins divided by the ridge lines.

Algorithm starts with setting an initial threshold, Ti. Morphological operation is performed for thresholding the intensity level globally. This operation begins with setting the structuring element that works like filter. These elements will filter out the background intensities in the image. The morphological operation is done to remove the dark and bright spot in the image and it is performed by using opening operation first which is like the erosion operation and then the closing operation likely to the dilation operation. When we are done with the morphological operation, we then computed the filtering operation which was done by convolving the morphologically smoothed image with a gaussian 3 × 3 kernel. Once this filtering was done, the gradient image operation was performed by finding the *x* and *y* gradient of the image and then found the gradient magnitude. The magnitude is the square root of the squared summation of the *x* and *y* gradient. Then, we selected a threshold for that gradient image. The threshold was set by computing the histogram operation of the maximum intensity value of the convolution operation of the morphological smoothed image.

**Fig. 5:**
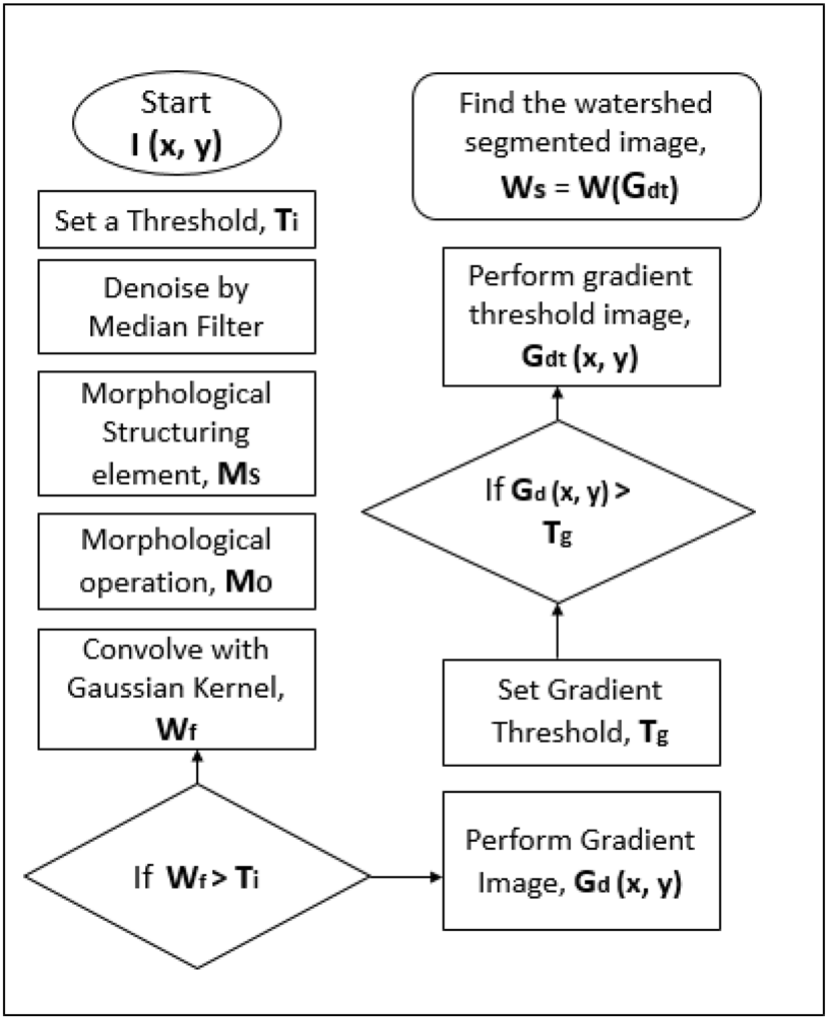
Flowchart of segmentation process using watershed algorithm. The initiation is accomplished by filtration, and then, the watershed transform is performed accordingly

Once we set the threshold value, the gradient image was compared with the threshold value. Upon the logical argument, we could find the gradient threshold image, *G*_dt_ (*x*, *y*). In that way, we computed the watershed labeled region of the tumor. The whole process is shown in Fig. 5 flowchart.

### Scale-invariant features transform (SIFT)

When we were done by segmenting the tumor region from the original image, we then performed the SIFT operation to match the segmented image by watershed with the ground truth image that is given in our benchmark dataset BRATS 2012. We computed the features from the two images, and to do that, we needed to perform the keypoints between the two images and then find the keypoint descriptor around that keypoints. We have shown the steps of this method in Fig. 2. Now, we will put down the steps in detail that we have performed for matching.

#### Scale-space peak finding

This is formed by convolution of the original image with the Gaussian functions of varying scales as shown in (1)

1 $$D(x,y,\sigma ) = L(x,y,k\sigma ) - L(x,y,\sigma )$$

where $L(x,y,k\sigma )$ and $L(x,y,\sigma )$ are the result of the convolution of the Gaussian functions with an input image $I(x,y).$

#### Keypoints detection

The pixel of the input is compared with the neighborhood pixels of above and below and thus found the local maxima and minima of the DoG (difference of Gaussian).

#### Keypoints localization

In this step, the edge points are eliminated due to their low-contrast points [18] by Taylor expansion as shown in (2)–(4),

2 $$D(x) = D + \frac{{\partial D^{T} }}{\partial x}x + \frac{1}{2}x^{T} \frac{{\partial^{2} D}}{{\partial x^{2} }}x$$

3 $$D(\hat{x}) = D + \frac{1}{2}\frac{{\partial D^{T} }}{\partial x}\hat{x}$$

4 $$\hat{x} = - \frac{{\partial^{2} D^{ - 1} }}{{\partial x^{2} }}\frac{\partial D}{\partial x}$$

#### Keypoint orientations

The orientation to each keypoint provides rotation invariance. The more invariance, the better it is. The magnitude and orientation are calculated for all pixels around the keypoints, and a histogram is created where 360 degrees of orientation are broken into 36 bins and each bin is proportional to the magnitude of gradient at that point.

#### Descriptor computation

The gradient magnitudes and orientations are sampled around the keypoint location. Then, these sampled values are illustrated with small arrows at each sample location [19].

We have introduced the overall steps of SIFT algorithm to create a new era in the image segmentation accuracy-check process. We implement a 16 × 16 array, and an 8-bin histogram is used for computing the keypoints orientation.

## Results and discussions

### 2D results

In method 1, we pass the input image through two steps of filtering and then apply watershed segmentation algorithm (WSA). Here, we use the trilateral filter and filtering the improvement factors is shown in Table 1.

**Table 1:** To initiate the watershed transform algorithm, input image needs to be filtrated by applying filter to improve the different factors of the image so that the algorithm can be compiled elucidately

Factors	Improved values
Original image variance	13,825,696.780211
Filtered variance	11,804,601.490946
Improvement factor	0.146184
Original standard deviation	79.256597
Filtered standard deviation	76.543790
Improvement factor	0.034228

**Fig. 6:**
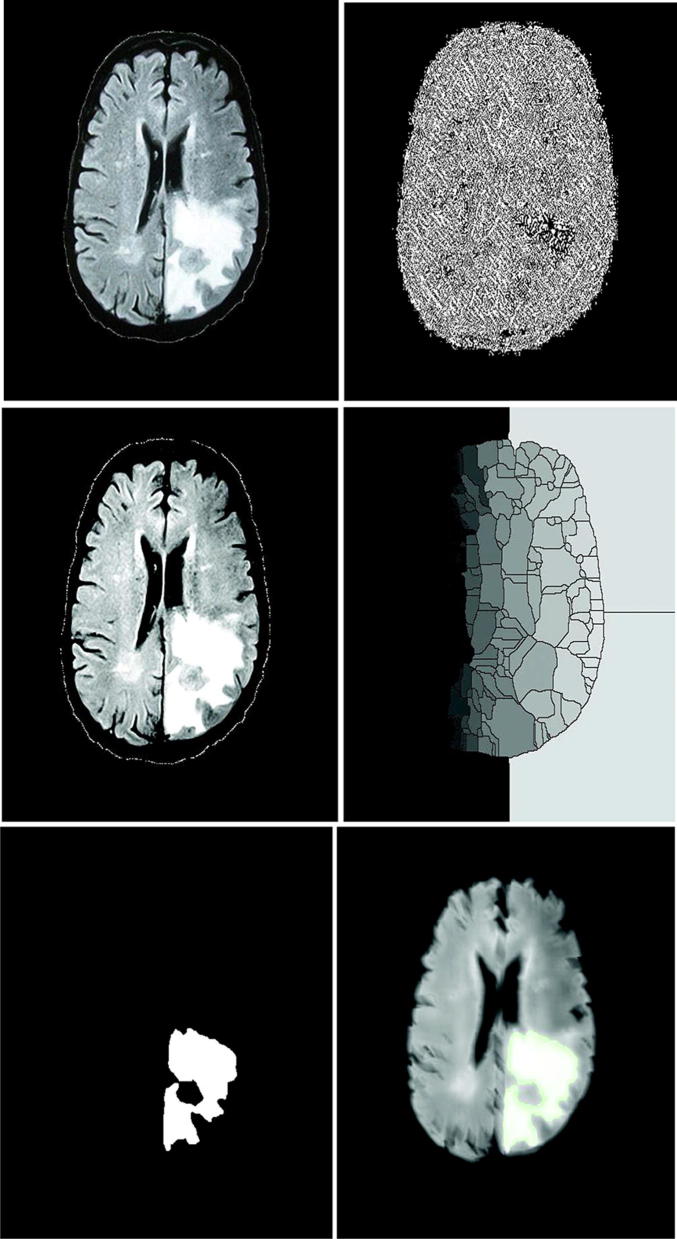
Experimental result of watershed segmentation (from left to right and top to bottom). Input MR brain tumor image, noise reduction and edge smoothing by trilateral filtering, de-noise image by a bilateral filter and reduction in impulse noise by median filtering, gradient watershed transform; white part refers to tumor segmentation using WSA after morphological operation, and blue contouring refers to tumor area detection using WSA

The watershed algorithm was tested on BRATS 2012 dataset, and we picked a single image to show the operation. We set the sigma value of the Gaussian kernel as 1 that removes the noise in the outer parts of the spectrum, and we set the initial threshold *T*_i_ = 10 for intensity threshold and *T*_g_ = 0.5 that is used for gradient threshold operation. We can observe from the fourth row of Fig. 6 that the tumor region is segmented from the earlier over-segmented watershed contour. We apply this transform to get the similar objectives out of the background. As we see from the gradient image, the region with less change in gray has lower gradient and gradient is higher in the neighbor boundary than the inside region. That is why a gradient structural element (size of 3 * 3) is used as the reference image. Then, we apply a morphological operation that includes opening and closing operation to get back the original one. In the dilation operation, original image is eroded first and then dilated. If the gradient image can fulfill the requirement of different shapes, then opening operation is performed as a union formation, and in this way, the whole image when goes through the filters, then the main image can be retained, and the noise is eliminated.

In the same way, closing operation is performed where the main image is firstly dilated. After this opening–closing operation, the original image is rendered, and thus, we get the segmented region as in the fourth row.

### 3D results

We analyze our algorithm for 3D MR images. We construct 3D image using 3D slicer, and we apply watershed algorithm. The input image is loaded in the software; then, we can see the directional images in three different windows. The volume model is built with the pre-chosen color. The first model is corpus callosum labeled color: green. The second model is frontal lobe white matter right labeled 17 (threshold 17:17), color: green. The third model is frontal lobe white matter left labeled 17 (threshold 17:17), color: green. Once we have segmented the target structure, we will use the Model Maker module to generate a surface volume, available in the full dropdown menu on the top toolbar. We also have a series of parameters within this section that we can modify, according to different parameters such as relief, color and luminosity. Once they are configured, three-dimensional image can be generated  by clicking “Create New Model Hierarchy” option. To better visualize the results, we can exclude the lower windows of multiplanar representations or change their distribution for a better correlation [20].

These tools are not exclusively circumscribed to the medical diagnosis field; they are also used in education, which in recent years has been changing traditional teaching methods for applications and technology that facilitate the interaction of students with the contents [21–24].

**Fig. 7:**
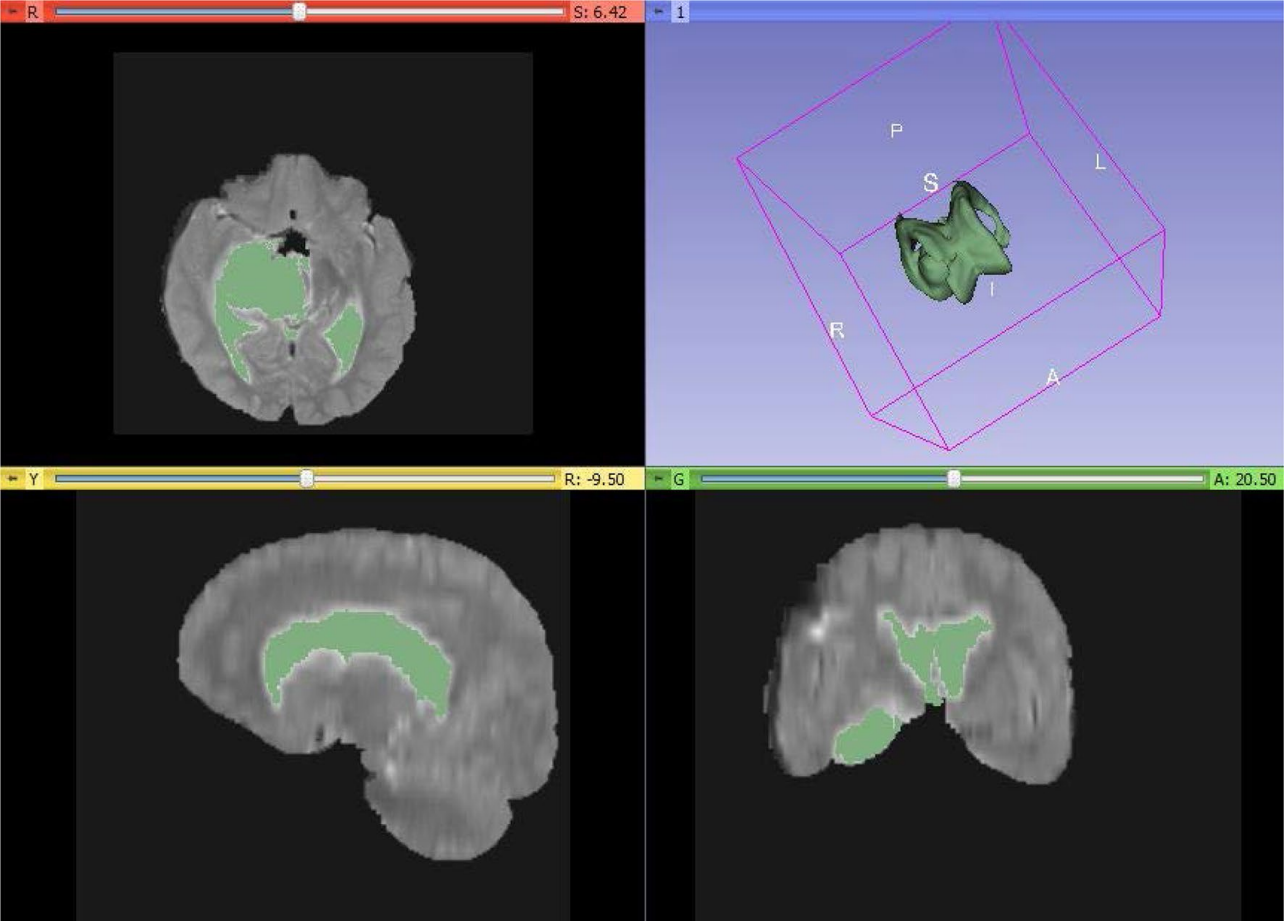
Experimental result of watershed segmentation (from top to bottom). all slices with the tumor in 3D, watershed result of brain tumor image (red slice only), tumor in 3D view only

So, we conclude that our algorithm works in 3D images also. Figure 7 shows that the tumor exists in the ventricles of the brain which is shown green color in the image. The image chosen for 3D segmentation was different than the image chosen for 2D operation.

### Sift results

**Fig. 8:**
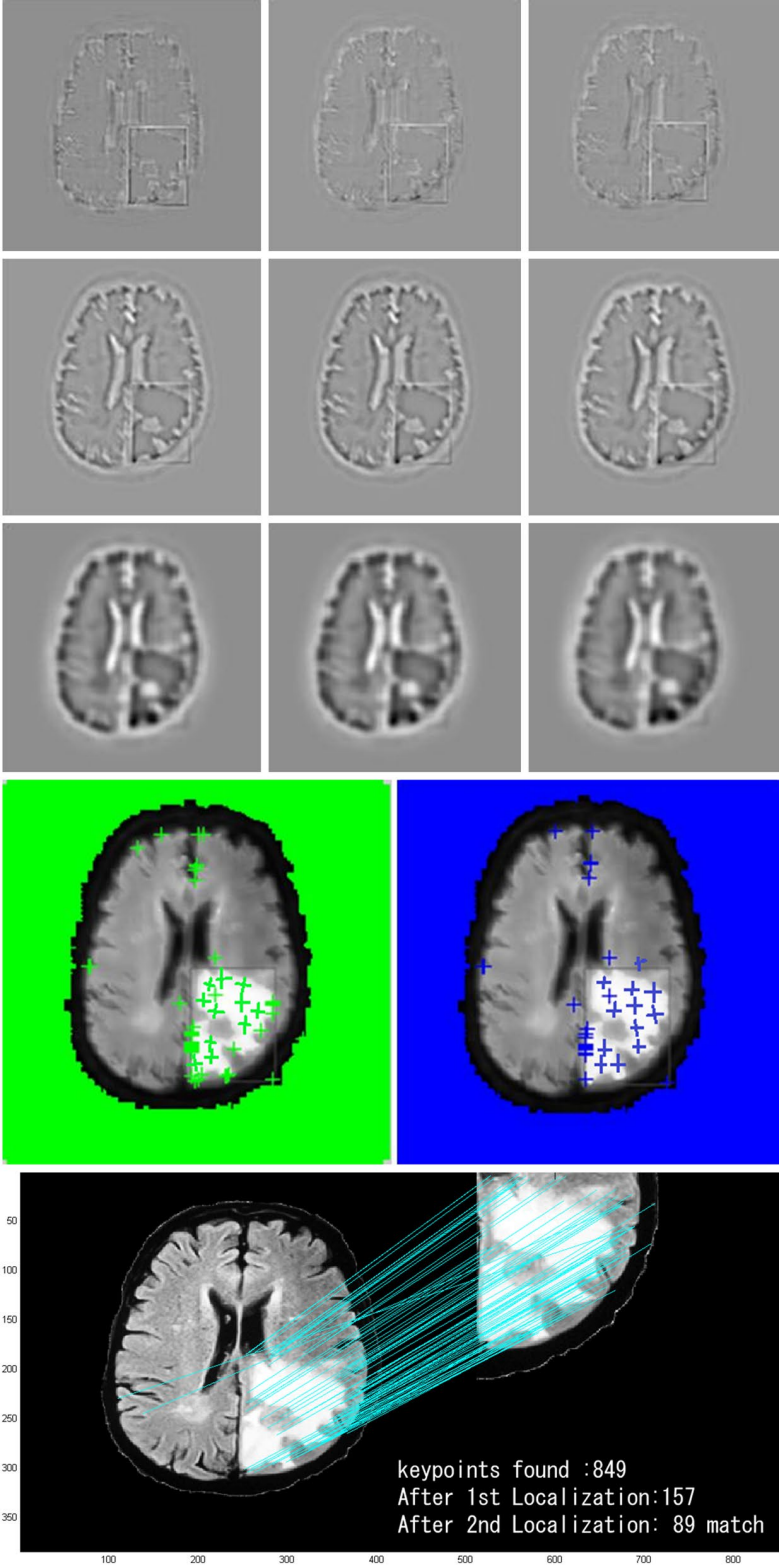
Experimental result of SIFT segmentation (from top to bottom). Finding features of input MR brain tumor image, finding keypoints; the density of green signs and the blue signs on the tumor area indicate the tumor region; keypoints are localized, and then, find matching and lastly find 89 matches between the input and the output image, thus confirming the tumor area from the binary image

We performed the SIFT algorithm not just to calculate the tumor area but also to justify the watershed segmentation. The watershed segmented image was matched with the ground truth image using this SIFT algorithm and that is why we named it as watershed-matching (WM) algorithm. We can observe from Fig. 7 that the input and output MR image that we use for watershed transform is also used for SIFT algorithm. According to the algorithm, we find the possible keypoints between input and output, and then, we discard the unreliable keypoints. Figure 8 indicates the confirmation of our complete algorithm that we name watershed-matching (WM) algorithm. For our experiments, we used T1-magnitude images. To create a training set of SIFT keypoints, firstly we find the features that carries the most relevant information. Then, we extract the keypoints to detect the tumor region. From Fig. 8, we can behold that the tumor region, which is our outcome, has the keypoints with high-density cluster (green/blue “+” sign). Lastly, the tumor is detected by the selection and matching of the extracted keypoints.

### Tumor area

From the last part of Fig. 8, the tumor area is calculated in the following ways:

$${\text{Image}} = \sum\limits_{l = 0}^{255} {} \sum\limits_{w = 0}^{255} {[x(0,{\text{white}}\;{\text{pixels}}) + x(1,{\text{black}}\;{\text{pixels}})]}$$

$${\text{White}}\;{\text{pixels}}\;{\text{count}}\quad W = \sum\limits_{l = 0}^{255} {\sum\limits_{w = 0}^{255} {[x(0,{\text{white}}\;{\text{pixels}})]} }$$

$${\text{Tumor}}\;{\text{size}} = [(W)]*0.264\;{\text{mm}}^{2}$$

where $$1\;{\text{Pixel}} = 0.264\;{\text{mm}}$$.

The tumor area is calculated to check the status of the tumor. As in our case, the area calculated was 269 for this MR image. So, it was in the initial stage. If the detected white pixels ≥ 500, then it will be in critical stage. To check that whether this pixel value calculation was correct or not, we computed the thickness calculation using the Freesurfer software and compared the result with our result. Though the result was not 100% correct, it gives the corrected result for most of the MRI test images.

The classical watershed algorithm segments the region perfectly, but for further clarification, we performed the SIFT algorithm to match the segmented tumor with the ground truth. So, our proposed algorithm provides two-step verification result.

## Conclusion

Brain tumor is treatable if it has been identified in the earliest stages of the disease. In this paper, we proposed and developed a novel approach for brain tumor segmentation and detection. Our main contribution consisted of modeling improved watershed algorithm with three steps of de-noising filtering and designing scale-invariant feature transform algorithm where the optimized features were selected. Traditional over segmentation problem could be minimized by our improved algorithm as the MR images are highly affected by noise and artifacts. We preprocessed the images using artifacts removal, median filter and trilateral filter for improving the segmentation quality. Due to this improved combination, our proposed method is far better than any single or other combination algorithms. To check the accuracy of our algorithm, we compared the result with the truth images and acquired 98.5% accuracy. Here, we also introduced status checking of the tumor. We calculated the area of the tumor and then set a decision rule to decide whether it is in a critical or initial stage. This status checking made our system more robust. Our framework can be used in the general application.

In future, we would use it not only for brain tumor segmentation but also for other applications like the bone tumor, lung tumor or other segmentation purposes. We will reduce several manual interactions. This will help the physicians to prosecute the further treatment process in advance to treat tumor patients.
