# Correction to: Two-step verification of brain tumor segmentation using watershed-matching algorithm

**DOI:** 10.1186/s40708-018-0089-7

**Published:** 2018-08-29

**Authors:** S. M. Kamrul Hasan, Mohiuddin Ahmad

**Affiliations:** 1 Department of Electrical and Electronic Engineering, Khulna University of Engineering Technology (KUET), Khulna, 9203 Bangladesh; 2 Present Address: Chester F. Carlson Center for Imaging Science, Rochester Institute of Technology (RIT), Rochester, NY 14623 USA

## Correction to: Brain Inf. (2018) 5:8 10.1186/s40708-018-0086-x

In the original publication of this article [1], the spelling of second author was incorrect. The correct name of the author should be Mohiuddin Ahmad. The original publication has been corrected.
